# Simulated Nitrogen Deposition Alters Disease Progression, Rhizosphere Soil Properties, and Microbiomes of *Pinus thunbergii* Infected by Pine Wood Nematode *Bursaphelenchus xylophilus*

**DOI:** 10.3390/plants15142200

**Published:** 2026-07-18

**Authors:** Chang-Jie Liao, Ting-Ting Jing, Si-Xi Lin, Hai-Jun Sun, Xiao-Lei Ding

**Affiliations:** Co-Innovation Centre for Sustainable Forestry in Southern China, Forestry and Grassland, College of Soil and Water Conservation, Nanjing Forestry University, Nanjing 210037, China; liaochangjie2023@163.com (C.-J.L.);

**Keywords:** pine wilt disease, inorganic N, soil microorganisms, nitrogen deposition, *Pinus thunbergii*

## Abstract

Pine wilt disease (PWD), caused by the pine wood nematode *Bursaphelenchus xylophilus*, is one of the most devastating forest diseases in Asia and Europe. In addition to causing rapid pine mortality, it can alter soil nutrient status and soil nitrogen transformation processes. At the same time, nitrogen (N) deposition is an important external nitrogen input to forest ecosystems and may further influence rhizosphere nitrogen dynamics under *B. xylophilus* infection; however, its effects on N forms and microbial community characteristics in the rhizosphere soil of infected pine trees remain poorly studied. Therefore, this study aimed to investigate the effects of simulated N deposition on PWD, rhizosphere soil chemical properties, and rhizosphere microbial community characteristics under *B. xylophilus* infection, by inoculating 4-year-old *Pinus thunbergii* seedlings with ddH_2_O (CK) and *B. xylophilus* (BX). For each inoculation condition, two simulated N deposition levels (N1: 50 mg N kg^−1^ dry soil; N2: 100 mg N kg^−1^ dry soil) and one control without N deposition (N0) were established. Generally, simulated N deposition significantly prolonged disease progression in *B. xylophilus*-infected pines, with mean times of 32, 43, and 46 days for BXN0, BXN1, and BXN2, respectively, and rhizosphere NO_3_^−^–N and NH_4_^+^–N contents were significantly higher in N1 and N2 for both the CK and BX groups. Under the same N deposition, BX treatment significantly enhanced the accumulation of NO_3_^−^–N but had a limited effect on NH_4_^+^–N. The microbial community analysis indicated that Ascomycota, Mortierellomycota, and Basidiomycota were the dominant fungal phyla across all experimental groups, while the *Talaromyces*, *Apiotrichum*, and *Aspergillus* genera showed significant abundance changes between the CK and BX groups. In addition, Actinobacteriota, Proteobacteria, and Acidobacteriota were the top bacterial phyla across all experimental groups, while the genera *Nocardioides* and *RB41* showed changes in relative abundance between the CK and BX groups. These findings suggest that short-term nitrogen deposition can influence the PWD process and modulate rhizosphere nitrogen dynamics and microbial community structure.

## 1. Introduction

Pine forests are an important forest type in temperate and subtropical regions around the world, and they play crucial ecological roles in carbon sequestration, soil stabilization, water regulation, and ecological restoration [1]. Pine wilt disease (PWD), caused by the pine wood nematode (*Bursaphelenchus xylophilus*), is one of the most destructive invasive diseases in many countries, killing millions of host pines worldwide and posing a severe threat to the ecosystem [2,3,4]. The wide dispersal of PWD is caused by vector insects and human-mediated timber transportation, leading to the decline in forest stand in infected areas [5]. In addition, outbreaks could significantly reduce forest carbon sequestration capacity and aboveground biomass, as well as change litter input patterns [6], as the rapid death of pines reduces root-derived carbon inputs and changes both the quantity and quality of litter. These changes can further affect soil physicochemical properties and nutrient cycling over time [7,8], and may also be associated with shifts in root-associated microbial communities across different belowground microhabitats, including changes in community structure and potential functions [9,10]. Therefore, the ecological impact of PWD expands from single-tree mortality to comprehensive ecological issues involving forest structure, carbon cycling, and soil system stability [11].

Previous studies have shown that *B. xylophilus* infection can simplify stand structure, thereby altering litter and root inputs and further inducing changes in soil nutrient status [12]—disturbances that soil nitrogen pools and related transformation processes are often sensitive to. Disease-driven changes may also affect the nitrogen mineralization and nitrification, thus raising the risk of soil fertility decline [13,14].

Soil microorganisms are major drivers of organic matter decomposition and nutrient transformation, and their diversity and community composition are closely linked to the maintenance of ecosystem functions [15]. Recent studies indicate that PWD can reshape rhizosphere and bulk-soil microbial communities during disease progression [16,17]; in particular, the diversity of fungal communities and the relative abundance of bacterial taxa may show marked changes. In some cases, the relative abundances of saprotrophic and potentially pathogenic groups increased, whereas those of symbiotic groups decreased [14,18,19]. Different belowground microhabitats, including the root endosphere, rhizosphere, and non-rhizosphere soil, may differ in microbial community diversity and composition in response to PWD infection. Since the rhizosphere serves as the key interface between host belowground processes and soil microorganisms, assessment of its microbiome is necessary [10].

Atmospheric nitrogen (N) deposition is an important source of external nitrogen input to forest ecosystems [20], and it can alter soil inorganic nitrogen availability, pH, and ion balance, and affect microbial community structure and nitrogen transformations [21]. The effects of N deposition depend on stand type and input strength; for instance, it may increase productivity in plantations, whereas natural forests may show negative responses under higher deposition levels [22]. Previous studies have shown that increased anthropogenic nitrogen emissions in China have significantly elevated forest N deposition levels over the past three decades [23], with the average total N deposition flux in its forests coming to 21.6 kg N ha^−1^ yr^−1^, indicating that the forest ecosystems have long been affected by external nitrogen inputs [24].

Taken together, PWD can cause a rapid decline in susceptible host pines and alter soil nutrient status and belowground microbial communities [7,8]. In the context of N deposition, it is ecologically important to examine how exogenous N inputs affect PWD development and rhizosphere soil N and microbial processes [25,26]. Nitrogen forms and input pathways involved in deposition are complex; thus, simulated N deposition enables assessment of how short-term external N input affects PWD progression and changes in rhizosphere soil nitrogen and microbial communities [27].

As mentioned above, many studies have examined the effects of N enrichment on forest soil microbial communities and nutrient responses [28,29]. However, whether external N input modifies PWD progression and the associated rhizosphere inorganic N dynamics and microbial responses during *B. xylophilus* infection remains poorly understood. To fill this knowledge gap, the progression of PWD and the dynamics of rhizosphere soil nutrients (including inorganic nitrogen) were monitored under simulated N deposition conditions, and high-throughput sequencing was performed to analyze the bacterial and fungal community structures and their potential functional changes in the rhizosphere soil. Our results may expand current understanding of the alterations in rhizosphere soil microbial communities and N forms of PWD-infected pine trees under N deposition.

## 2. Results

### 2.1. Effects of Simulated N Deposition on PWD Progression

**Figure 1:**
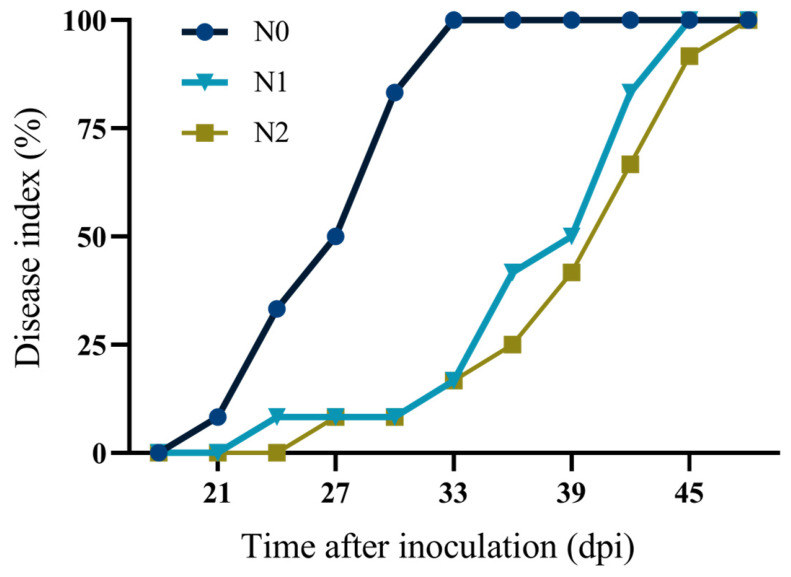
Temporal dynamics of disease severity index in *Pinus thunbergii* plants inoculated with *Bursaphelenchus xylophilus* under simulated N deposition conditions. Values are presented as means of three biological replicates; dpi, days post-inoculation.

After *B. xylophilus* inoculation, the DSI in BXN0 (inoculation of *B. xylophilus* without simulated N deposition) treatment continued to increase from 21% and reached 100% at 33 dpi (Figure 1), while that of pine trees under BXN1 and BXN2 reached 100% at 48 dpi and 45 dpi, respectively, demonstrating that pine trees under BXN0 without simulated N deposition showed the most rapid disease progression, followed by BXN1 and BXN2 (Figure 1). Statistically, the N1 and N2 treatments significantly delayed the mortality time of pines infected by *B. xylophilus* (*p* < 0.05) compared to BXN0 and were not significantly different from each other (Table S1, Figure 1).

### 2.2. Effects of Simulated N Deposition on Rhizosphere Soil Chemical Properties

**Figure 2:**
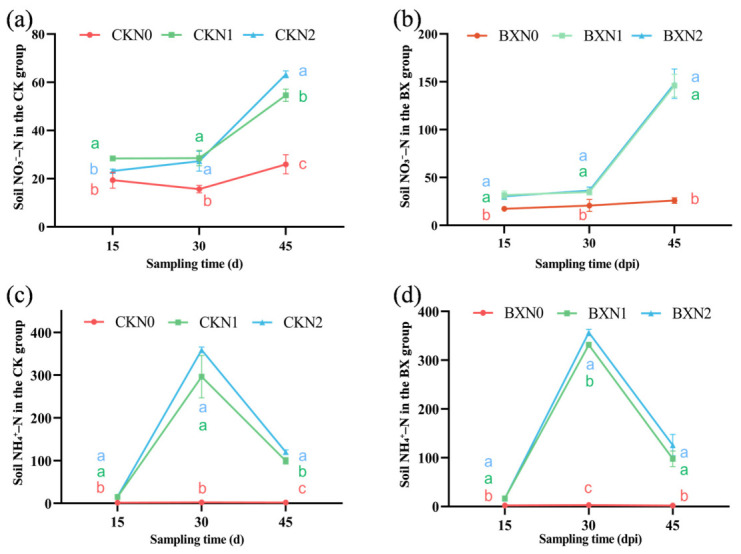
Dynamics of soil inorganic nitrogen under pine wood nematode inoculation and simulated N deposition during disease progression. Values are presented as means ± SE (*n* = 3). Different lowercase letters indicate significant differences among simulated conditions within the same inoculation condition and the same sampling time (*p* < 0.05). CK, control without *B. xylophilus* inoculation; BX, treatment with *B. xylophilus* inoculation; dpi, days post-inoculation. (**a**) NO_3_^−^–N in the CK group; (**b**) NO_3_^−^–N in the BX group; (**c**) NH_4_^+^–N in the CK group; and (**d**) NH_4_^+^–N in the BX group.

The initial soil chemical properties and those of the rhizosphere of the six experimental groups at 45 dpi are presented in Table 1. Rhizosphere NO_3_^−^–N and NH_4_^+^–N contents were higher in N1 and N2 than in N0, regardless of *B. xylophilus* inoculation (Figure 2). NO_3_^−^–N accumulation was mainly observed at 45 dpi (Figure 2a,b; Table S1). Specifically, its contents at 45 d were 2.1 and 2.4 times higher in CKN1 and CKN2 than in CKN0 (*p* < 0.05; Figure 2a), and approximately 5.6 and 5.7 times higher in BXN1 and BXN2 than BXN0 (*p* < 0.05; Figure 2b), respectively. Further comparisons between CK and BX treatments showed that the latter significantly enhanced the accumulation of NO_3_^−^–N under both N1 and N2 (Figure 2a,b; Table S1), but no significant difference was observed between CKN0 and BXN0.

In contrast, NH_4_^+^–N responded rapidly to simulated N deposition and reached its maximum at 30 dpi, followed by a decline at 45 dpi in all treatments (Figure 2c,d; Table S1). At 30 dpi, its contents in CKN1 and CKN2 were approximately 117.6 and 142.5 times higher than in CKN0 (Figure 2c); meanwhile, BXN1 and BXN2 showed a 121.4- and 130.7-fold change compared with BXN0, respectively (Figure 2d). No significant difference was observed between CK and BX regardless of N deposition (Table S1).

Soil pH also increased under simulated N deposition, whereas SOM and AP did not show consistent treatment responses (Table 1, Table S1). At 45 dpi, soil pH was significantly higher in CKN1 and CKN2 than in CKN0, as well as in BXN2 compared to that in BXN0 and BXN1 (*p* < 0.05).

### 2.3. Microbial α-Diversity in Rhizosphere Soil Under Simulated N Deposition

**Figure 3:**
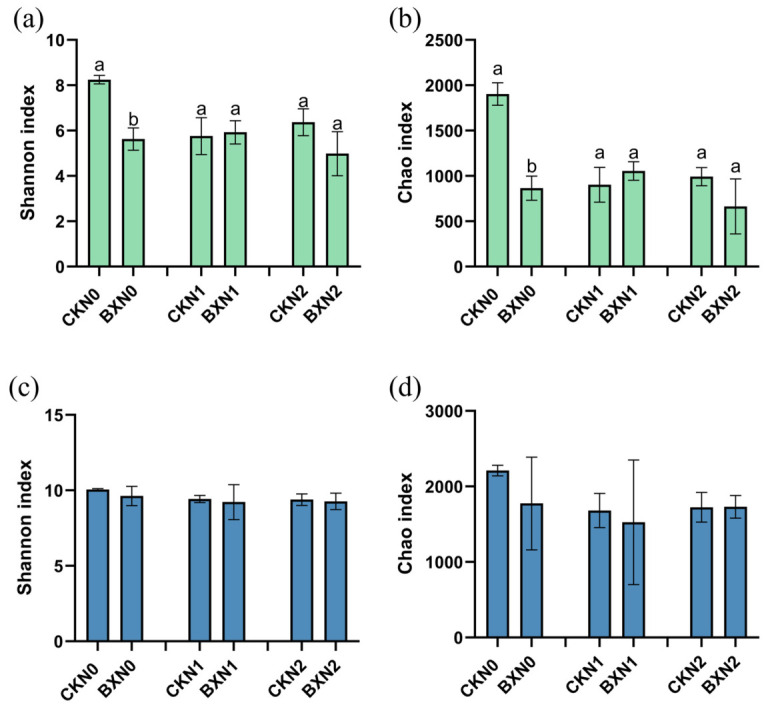
**α**-diversity indices of rhizosphere fungal (**a**,**b**) and bacterial (**c**,**d**) communities under different treatments. Bars represent means ± SE (*n* = 3). Different lowercase letters, indicate significant differences among treatments (*p* < 0.05).

A total of 13,708 bacterial and 8563 fungal ASVs was obtained from all soil samples. Good’s coverage exceeded 99.0% for all samples, indicating sufficient sequencing depth for α-diversity analysis. Fungal Shannon and Chao1 indices were significantly lower in BXN0 than in CKN0 (*p* < 0.05; Figure 3a,b); however, no significant differences were detected between CK and BX under N1 or N2. Additionally, fungal α-diversity indices in CKN0 were notably higher than those in the other treatments (Figure 3a,b). Bacterial α-diversity indices did not differ significantly among all treatments (Figure 3c,d).

### 2.4. Microbial β-Diversity in Rhizosphere Soil Under Simulated N Deposition

Under the same simulated N deposition condition, pairwise comparisons based on Bray–Curtis distances showed significant variations in fungal community composition between CKN2 and BXN2 only (*p* < 0.05; Table 2). Significant variations in the composition of bacterial communities were found between CK and BX under both N0 (*p* < 0.01) and N1 (*p* < 0.001), whereas no significant difference was observed under N2 (*p* > 0.05; Table 2).

### 2.5. Microbial Community Composition

**Figure 4:**
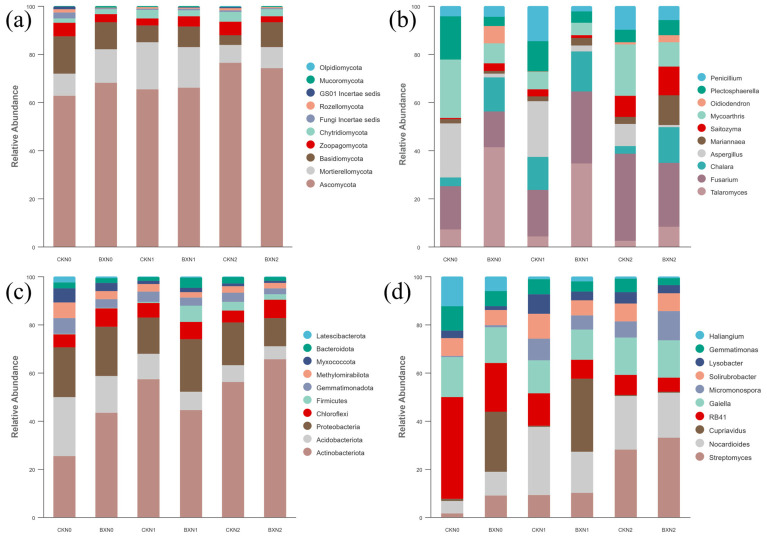
Relative abundances of the top 10 dominant fungal and bacterial across treatments. Stacked bar plots show the mean relative abundance (%) of each taxon (mean for three replicates). Panels show fungal communities at the phyla (**a**) and genera (**b**) levels and bacterial communities at the phyla (**c**) and genera (**d**) levels.

A total of 19 fungal phyla were identified, among which Ascomycota was the most abundant, followed by Mortierellomycota and Basidiomycota (Figure 4a, Table S2). Statistically, Mortierellomycota was significantly more abundant in CKN0 than in BXN0, as well as in CKN1 compared to BXN1 (*p* < 0.05; Table S2). Meanwhile, Basidiomycota was significantly enriched in BXN2 only (*p* < 0.05; Table S2).

A total of 695 fungal genera were identified, accounting for 68.1% of the total sequences; the remaining sequences were unclassified at the genus level (Figure 4b; Table S2). Significant differences between CK and BX were mainly observed in *Talaromyces*, *Apiotrichum*, and *Aspergillus*. *Aspergillus* showed higher relative abundance in CK than in BX under N0, N1, and N2 treatments (*p* < 0.05; Table S2), and *Talaromyces* was significantly higher in BXN0 and BXN1 than in CKN0 and CKN1 (*p* < 0.05; Table S2), respectively. Meanwhile, *Apiotrichum* showed the higher relative abundance in CKN0 than in BXN0. The abundances of other dominant genera like Fusarium and Chalara were not significantly changed after BX treatment across all N deposition levels.

A total of 37 bacterial phyla were identified, among which Actinobacteriota was the most abundant, followed by Proteobacteria and Acidobacteriota (Figure 4c; Table S3). The relative abundance of Proteobacteria differed significantly between CKN2 and BXN2, and that of Acidobacteriota was enriched in CKN0 and CKN1 in comparison with BXN0 and BXN1 (*p* < 0.05; Table S3).

A total of 545 bacterial genera were identified, accounting for 82.3% of the total sequences; the remaining sequences were unclassified at the genus level (Figure 4d; Table S3). Significant differences between CK and BX were mainly observed in *Nocardioides* and *RB41*. *Nocardioides* were significantly more abundant in BXN0 than in CKN0; however, under N1, it was significantly more abundant in CKN1 than in BXN1 (*p* < 0.05; Table S3). *RB41* was highly enriched in CKN0 and CKN1, in comparison with BXN0 and BXN1, respectively (*p* < 0.05; Table S3).

### 2.6. Potential Functional Structures of Microbial Communities

**Figure 5:**
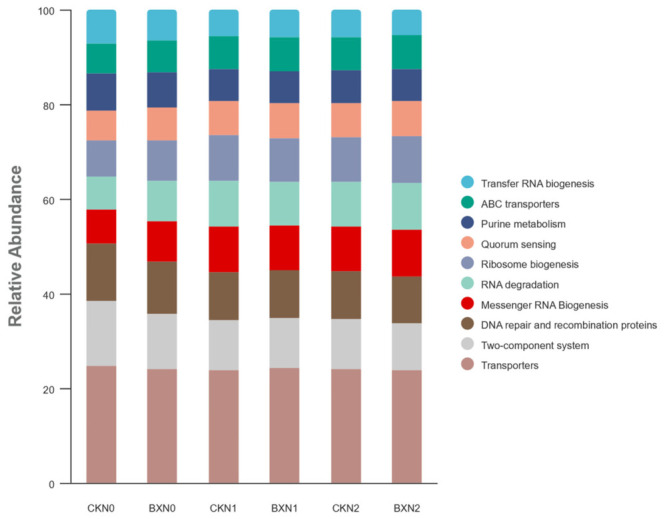
Functional profiles of rhizosphere bacterial communities under different treatments, showing the relative abundances of functional categories predicted by Tax4Fun (mean for three replicates).

Tax4Fun prediction identified 390 functional categories in the bacterial community, of which the most abundant was transporters, followed by two-component system, DNA repair and recombination proteins, etc. (Figure 5). No significant differences were detected in any of the predicted functions between CK and BX under the same simulated N deposition level (*p* > 0.05; Table S4). Functional analysis of fungal communities indicated that less than 20% of fungal species (ASVs) were assigned to any trophic mode; thus, we did not perform further analysis to avoid misinterpretation.

## 3. Discussion

Human activities have significantly increased the deposition of reactive nitrogen (N) in terrestrial ecosystems, with profound implications for global ecosystems and biodiversity [30]. Furthermore, nitrogen deposition can substantially alter the structure and function of forest ecosystems [23]: studies have revealed that it not only directly affects soil physicochemical properties and nutrient cycling processes, but also modulates the assembly and functional expression of the rhizosphere microbial communities [31,32]. In this study, we found that simulated nitrogen deposition treatments could significantly delay, but not prevent, the mortality of black pine seedlings (Figure 1), which is consistent with previous studies showing that moderate nitrogen addition can improve host nutritional status and photosynthesis, thereby increasing plant resistance to pathogens and slowing disease progression [33]. This extended disease progression may facilitate disease monitoring, early diagnosis, and integrated management strategies.

In addition, rhizosphere soil NO_3_^−^–N content increased in all treatments and exhibited significantly higher levels in the black pine rhizosphere soil under BX treatment; in contrast, soil NH_4_^+^–N content increased initially and decreased at 45d and no significant difference was observed between the BX and CK treatments (Figure 2). These results indicate that PWD infection accelerated the conversion of soil NH_4_^+^–N to NO_3_^−^–N, which is consistent with the fundamental principles of soil nitrogen cycling [34]: under aerobic conditions, soil nitrifying microorganisms can oxidize NH_4_^+^ to NO_2_^−^ and subsequently to NO_3_^−^. Therefore, although deposition increases the availability of nitrogen in the rhizosphere soil, PWD may further modulate nitrogen transformation and absorption by inhibiting the host and its mycorrhizal symbiotic system [35].

Previous studies have shown that PWD can reshape root-associated and rhizosphere microbial communities [10,14]. Moreover, nitrogen deposition itself can alter soil microbial community structure [36]. In this study, *B. xylophilus* infection only significantly reduced fungal α-diversity under N0. Another study indicated PWD did not alter the diversity of rhizosphere bacteria, but significantly decreased the richness of rhizosphere fungi [14]. Similarly, the richness and diversity of root-associated fungi were significantly lower in PWD-disturbed stand than that of undisturbed stand [8]; thus, PWD usually reduces fungal α-diversity but has a limited effect on bacterial α-diversity. In addition, fungal and bacterial community compositions changed only in N2, and N0 and N1 (Table 2), respectively. A relevant study focusing on the response of soil microbial communities in coniferous forests to nitrogen deposition claimed nitrogen addition significantly influenced the β-diversity of fungal communities more than that of bacterial communities [37]; however, we found that short-term simulated N deposition has the potential to alter the community compositions of both fungi and bacteria under BX treatment.

Furthermore, we found that the relative abundance of *RB41* was significantly higher in CK than BX under both N0 and N1 conditions, similarly to a study on the inter-root microbial community on the inhibition of anthracnose in peppers [38]. *RB41* was also highly enriched in root soil collected from highly resistant pepper inoculated with anthracnose compared to disease-susceptible pepper *RB41*, which belongs to the phylum Acidobacteria and is involved in soil carbon cycling and nitrogen transformation; its higher abundance may reflect enhanced soil nutrient supply capacity [39,40]. Therefore, *RB41* may represent a potential beneficial indicator microorganism associated with plant disease resistance and soil health, and its enrichment in healthy or disease-resistant soils may contribute to maintaining soil microecological stability and nutrient cycling functions. In addition, the relative abundance of *Nocardioides* was significantly higher under BX treatment at N0 deposition than at N1, suggesting that nitrogen deposition may drive a shift in the dominance of microbes like *Nocardioides* [41], which is capable of secreting cellulase to enhance the decomposition of complex organic matter and the release of nutrients to promote plant resistance [42]. The higher abundance of this bacteria could possibly promote soil fertility and enhance plant growth and resistance.

Additionally, our findings revealed that the relative abundance of *Talaromyces* was enriched in BXN0 and BXN1 while *Aspergillus* was enriched in CK. The former contains species like *T. flavus*, *T. albobiverticillius*, *T. pinophilus*, and *T. purpurogenus*, which have the potential for promoting plant growth and tolerance to various stresses [43,44,45], while a few of them were recognized as plant pathogens like *T. domesticus* and *T. minioluteus* [46,47]. Other *Talaromyces* species are also capable of producing mycotoxins in food products (*T. islandicus*, *T. radicus*, and *T. rugulosus*) [48], and some have been postulated as excellent enzyme producers for plant biomass applications [49,50]. *Aspergillus* species populations are diverse and some of them are known as fermentation agents and degraders of agricultural products (*A. niger* and *A. flavus*) [51,52]; species like *A. flavus*, *A. oryzae*, *A. niger*, and *A. carbonarius* are also reportedly associated with plant disease [53,54]. In addition, *A. conicus* and *A. terreus* were identified as plant-growth fungi [55,56]. PWD infection clearly changed the abundance of these two genera and potentially affected their contribution to plant growth and biomass valorization. Furthermore, *Fusarium* and *Chalara* were two other abundant genera found in this study. However, short-term N deposition and BX treatment had no significant effects on these two fungal species. *Fusarium* species represent a vast and diverse group of filamentous ascomycete fungi mostly known as plant pathogens usually found on the lower part of plant stems and soil-dwelling saprotrophs (*F. solani*, *F. oxysporum*, and *F. graminearum*) [57,58]. Meanwhile, *F. oxysporum* has also shown nematicidal activity against pine wood nematode [59]. *Chalara* species live mostly as litter saprotrophs; many of them are found on coniferous litter (*C. longipes* and *C. hyalocuspica*) [60], and a few of them have also been identified as plant pathogens such as *C. elegans*, which causes root-rot disease on tomato [61] and lettuce [62], as well as *C. fraxinea*, which causes dieback on Fraxinus excelsior [63].

Functional prediction of rhizosphere bacteria communities indicated that the potential functions of rhizosphere bacteria across different treatments were concentrated in pathways related to metabolism and membrane transport. In a study of the rhizosphere microbiome of *Tamarix* under salt stress, metabolism-related genes consistently accounted for the highest proportion, while membrane transporters (such as ABC transporters) and replication–repair systems were changed in response to environmental stress [64]. It is possible that the aforementioned functional categories can be found in rhizosphere microbiomes from different host trees in response to environmental changes like pathogen infection and external stress.

## 4. Materials and Methods

### 4.1. Experimental Design

Four-year-old potted *P. thunbergii* plants were used in this experiment, which were grown in 1.5-gallon (approximately 5.68 L) plastic pots, each filled with approximately 1.5 kg of yellow-brown soil collected from the 0–20 cm layer of the Nanjing Forestry University Arboretum (32°04′ N, 118°49′ E). Plant residues and stones were removed in the laboratory, and the soil was sieved before use. A 2 × 3 factorial design was used, with *B. xylophilus* inoculation condition as one factor, including a control without *B. xylophilus* inoculation (CK) and treatment with *B. xylophilus* inoculation (BX), and simulated N deposition condition as the other factor (N0, N1, and N2; see Section 2.2). Thus, six experimental groups (CKN0, CKN1, CKN2, BXN0, BXN1, and BXN2) were used in this study, with three biological replicates per treatment, resulting in 18 pots. The pot experiment was conducted in a research greenhouse at Nanjing Forestry University.

### 4.2. Simulated N Deposition Conditions

Urea [CO(NH_2_)_2_; 46% N], which is the mass percentage of nitrogen in urea, was used as the N source, and one control treatment without N deposition (N0) and two simulated N deposition levels (N1, 50 mg N kg^−1^ dry soil; N2, 100 mg N kg^−1^ dry soil) were established. Nitrogen was applied once, 24 h before nematode inoculation. Urea was dissolved in 200 mL of deionized water and evenly applied to the soil surface of each pot; the N0 control received only an equal volume of ddH_2_O. After application, all pots were maintained under the same watering regime.

### 4.3. Pine Wood Nematode Inoculation and Disease Assessment

The *Bursaphelenchus xylophilus* strain used for inoculation was obtained from the Pathology Laboratory of Nanjing Forestry University, and nematodes were extracted using the Baermann funnel method to prepare the inoculum suspension [65]. Pine wood nematode inoculation was performed 24 h after N application: a wound was created on a vigorous branch of each plant, and 4000 nematodes were inoculated using a 1.5 mL microcentrifuge tube inoculation method. Non-inoculated controls received an equal volume of ddH_2_O as a mock inoculation. The disease symptoms were assessed every 3 d, and disease severity index was scored on a 0–4 scale based on previous methods: DSI = Σ (disease grade × number of plants at that grade)/(maximum grade × total number of plants) × 100% [66].

### 4.4. Rhizosphere Soil Sampling

Rhizosphere soil samples were collected from the pots 15, 30, and 45 days post-inoculation (dpi). In each pot, three sampling holes were selected with 120° intervals around the stem, and the soils in those holes were collected by a 16 mm diameter soil corer and mixed together. Immediately after sampling, the sampling holes were backfilled with adjacent soil from the same pot.

### 4.5. Soil Chemical Analysis

The remaining soil was air-dried, ground, and passed through a 2 mm sieve before measuring pH, soil organic matter (SOM), and available phosphorus (AP). NH_4_^+^–N and NO_3_^−^–N were extracted with 2 mol L^−1^ KCl and determined by UV–visible spectrophotometry (Shimadzu UV-1800, Kyoto, Japan) [67], soil pH was measured with a pH meter (Mettler Toledo, Shanghai, China) at a soil: water ratio of 1:2.5, SOM was determined by the potassium dichromate oxidation method, and AP was extracted with 0.5 mol L^−1^ NaHCO_3_ and measured using the molybdenum antimony colorimetric method [68].

### 4.6. Microbial DNA Extraction, Amplification, and Sequencing

Total soil DNA was extracted from the fresh rhizosphere soil samples collected 45 d after the nematode inoculation by Novogene (Beijing, China) using a soil genomic DNA extraction kit (TIANGEN BIOTECH, Beijing, China; DP336) according to the manufacturer’s instructions. The V4–V5 region of the bacterial 16S rRNA gene and the fungal ITS1 region were amplified using primers 515F/907R (GTGCCAGCMGCCGCGGTAA/CCGTCAATTCCTTTGAGTTT) and ITS1F/ITS2R (GGAAGTAAAAGTCG TAACAAGG/GCTGCGTTCTTCATCGATGC), respectively. Each PCR reaction contained 15 μL of Phusion High-Fidelity PCR Master Mix (New England Biolabs, Ipswich, MA, USA), 0.2 μM of each primer, and 10 ng of template DNA, and PCR amplification was performed under the following conditions: initial denaturation at 98 °C for 1 min; 30 cycles of 98 °C for 10 s, 50 °C for 30 s, and 72 °C for 30 s; and a final extension at 72 °C for 5 min. PCR products were checked by 2% agarose gel electrophoresis. After library construction, libraries were quantified using Qubit 3.0 fluorometer (Thermo Fisher Scientific, Waltham, MA, USA) and qPCR system (Life Technologies, Carlsbad, CA, USA) and then sequenced on the Illumina NovaSeq 6000 platform (Illumina, Inc., San Diego, CA, USA) using paired-end sequencing with 250 bp reads (PE250).

### 4.7. Sequence Processing and Functional Prediction

Raw sequences were processed using QIIME 2 (v2022.02), for quality filtering, chimera removal, denoising, amplicon sequence variant (ASV) generation, and taxonomic assignment [69,70]. Bacterial and fungal sequences were taxonomically assigned using the SILVA 138.1 and UNITE v9.0 databases, respectively [71,72]. Potential bacterial functions were predicted using Tax4Fun based on taxonomic mapping [73]; these were used only to compare profiles among treatments and should not be interpreted as direct measurements of functional processes.

### 4.8. Statistical Analysis

One-way ANOVA followed by Tukey’s HSD test was used to compare the PWD time to death and chemical properties of rhizosphere soil among the three N deposition treatments. The rhizosphere soil properties were also compared between CK and BX under the same simulated N deposition condition by employing the independent-samples *t*-test.

Before α- and β-diversity analyses, the ASV table was rarefied to the minimum sequencing depth across samples. Microbial α-diversity indices, including Shannon index, Chao1 richness estimator, and Good’s coverage, were compared between CK and BX under the same simulated N deposition level employing the independent-samples *t*-test. Microbial β-diversity was assessed based on Bray–Curtis distances calculated from the ASV abundance matrix, and the distributions of pairwise Bray–Curtis distances between CK and BX under the same N level were compared using a one-way ANOVA followed by Tukey’s HSD test. The relative abundances of dominant microbial taxa were compared between CK and BX under the same simulated N deposition condition employing the independent-samples *t*-test.

Statistical analyses were performed using SPSS 26.0 (IBM Corp., Armonk, NY, USA) and the Novogene Cloud Platform (Novogene Co., Ltd., Beijing, China).

## 5. Conclusions

Generally, N deposition simulation did not alter the ultimate mortality rate of *P. thunbergii* infected with PWD, but significantly delayed disease progression, an effect that may be closely associated with elevated rhizosphere inorganic nitrogen concentrations (particularly NO_3_^−^–N) and shifts in nitrogen form distribution. At the microbial level, the combined effects of BX treatment and nitrogen deposition led to taxon-specific shifts in the fungal and bacterial communities of the rhizosphere soil. These findings suggest that short-term nitrogen deposition can influence the PWD process by modulating rhizosphere nitrogen dynamics and microbial community structure; however, its long-term effects warrant further investigation.

## Figures and Tables

**Table 1:** Chemical properties of initial and rhizospheric soil in the six experimental groups at 45 dpi.

Indicator	Initial Soil	CKN0	CKN1	CKN2	BXN0	BXN1	BXN2
NO_3_^−^–N (mg kg^−1^)	22.10 ± 3.95	26.00 ± 2.29 cA	54.63 ± 1.45 bB	63.33 ± 0.82 aB	26.00 ± 1.71 bA	146.12 ± 6.82 aA	148.09 ± 8.85 aA
NH_4_^+^–N (mg kg^−1^)	27.18 ± 1.73	2.17 ± 0.11 cA	99.64 ± 4.33 bA	120.04 ± 2.88 aA	1.80 ± 0.27 bA	97.85 ± 9.15 aA	125.94 ± 12.61 aA
Soil pH	6.31 ± 0.05	6.29 ± 0.03 cA	7.10 ± 0.13 bA	7.89 ± 0.19 aA	6.38 ± 0.03 cA	6.70 ± 0.07 bA	7.75 ± 0.06 aA
SOM (g kg^−1^)	123.75 ± 3.11	93.30 ± 5.26 aA	97.85 ± 2.63 aA	102.41 ± 4.55 aA	87.99 ± 2.74 aA	100.13 ± 5.26 aA	97.85 ± 3.94 aA
AP (mg kg^−1^)	45.63 ± 0.19	58.34 ± 0.85 aA	57.98 ± 1.20 aA	55.45 ± 0.18 aA	54.25 ± 0.57 aA	54.47 ± 1.46 aA	53.53 ± 1.85 aA

Note: Values are means ± SE (*n* = 3). Lowercase letters indicate significant differences among N0, N1, and N2 within the same inoculation condition at 45 dpi (*p* < 0.05). Uppercase letters indicate significant differences between CK and BX under the same simulated N deposition condition at 45 dpi (*p* < 0.05). CK, control without *B. xylophilus* inoculation; BX, treatment with *B. xylophilus* inoculation; N0, control without simulated N deposition; N1, low simulated N deposition treatment; N2, medium simulated N deposition treatment.

**Table 2:** Pairwise comparisons of microbial community composition based on Bray–Curtis distances between CK and BX under the same simulated N deposition level.

Community	Comparison	*p*-Value
Fungi	CKN0 vs. BXN0	0.0859
Fungi	CKN1 vs. BXN1	0.6955
Fungi	CKN2 vs. BXN2	0.0332 *
Bacteria	CKN0 vs. BXN0	0.0015 **
Bacteria	CKN1 vs. BXN1	0.0005 ***
Bacteria	CKN2 vs. BXN2	0.1176

* *p* < 0.05; ** *p* < 0.01; *** *p* < 0.001.

## Data Availability

Strains are available upon request. The authors affirm that all data necessary for confirming the conclusions of the article are present within the article, figures, and tables.

## Supplementary Materials

The following supporting information can be downloaded at https://www.mdpi.com/article/10.3390/plants15142200/s1, Table S1: Rhizosphere soil chemical properties at different disease stages under different inoculation treatments and simulated N deposition level; Table S2: Relative abundances (%) of selected fungal taxa discussed in the manuscript; Table S3: Relative abundances (%) of selected bacterial taxa discussed in the manuscript; Table S4: Relative abundances (%) of selected predicted bacterial KEGG level-3 functional categories.
