# Supplementary material for: Simulated Nitrogen Deposition Alters Disease Progression, Rhizosphere Soil Properties, and Microbiomes of Pinus thunbergii Infected by Pine Wood Nematode Bursaphelenchus xylophilus
Source: Plants (Basel). 2026 Jul 18;15(14):2200. doi: 10.3390/plants15142200 (PMC13416299; doi:10.3390/plants15142200)
Supplement: Supplementary file 1 [file plants-15-02200-s001.zip › plants-4362236-supplementary.pdf]

Supplementary Tables

Table S1. Rhizosphere soil physicochemical properties at different disease stages under different inoculation treatments and simulated N deposition levels

Property	Stage	CKN0	CKN1	CKN2	BXN0	BXN1	BXN2
pH	15 dpi	6.95 ± 0.02bA	8.03 ± 0.22aA	8.48 ± 0.02aA	6.24 ± 0.08bB	8.41 ± 0.02aA	8.41 ± 0.01aA
pH	30 dpi	6.46 ± 0.06cA	7.27 ± 0.19bA	8.41 ± 0.10aA	6.43 ± 0.07cA	6.90 ± 0.10bA	8.19 ± 0.12aA
pH	45 dpi	6.29 ± 0.03cA	7.10 ± 0.13bA	7.89 ± 0.19aA	6.38 ± 0.03cA	6.70 ± 0.07bA	7.75 ± 0.06aA
NO_3^- − N (mg kg^{-1})	15 dpi	19.41 ± 1.90bA	28.42 ± 0.59aA	23.21 ± 0.46bB	17.35 ± 1.51bA	31.78 ± 2.39aA	30.40 ± 1.95aA
NO_3^- − N (mg kg^{-1})	30 dpi	15.71 ± 0.89bA	28.59 ± 1.86aA	27.28 ± 2.37aB	20.78 ± 3.60bA	34.93 ± 1.77aA	36.34 ± 2.14aA
NO_3^- − N (mg kg^{-1})	45 dpi	26.00 ± 2.29cA	54.63 ± 1.45bB	63.33 ± 0.82aB	26.00 ± 1.71bA	146.12 ± 6.82aA	148.09 ± 8.85aA
NH_4^+ − N (mg kg^{-1})	15 dpi	1.80 ± 0.04bA	15.22 ± 1.16aA	15.99 ± 0.91aA	2.07 ± 0.10bA	16.53 ± 0.19aA	16.11 ± 0.30aA
NH_4^+ − N (mg kg^{-1})	30 dpi	2.52 ± 0.04bA	296.39 ± 28.66aA	359.23 ± 3.70aA	2.73 ± 0.25cA	331.50 ± 3.13bA	356.74 ± 3.70aA
NH_4^+ − N (mg kg^{-1})	45 dpi	2.17 ± 0.11cA	99.64 ± 4.33bA	120.04 ± 2.88aA	1.80 ± 0.27bA	97.85 ± 9.15aA	125.94 ± 12.61aA
AP (mg kg^{-1})	15 dpi	56.24 ± 0.86aA	52.95 ± 0.74bA	55.28 ± 0.45abA	57.07 ± 1.62aA	52.38 ± 1.95abA	49.27 ± 0.66bB
AP (mg kg^{-1})	30 dpi	49.94 ± 1.52aA	46.02 ± 1.03aA	47.71 ± 0.50aA	42.83 ± 1.13aB	44.43 ± 0.39aA	43.58 ± 0.96aB
AP (mg kg^{-1})	45 dpi	58.34 ± 0.85aA	57.98 ± 1.20aA	55.45 ± 0.18aA	54.25 ± 0.57aB	54.47 ± 1.46aA	53.53 ± 1.85aA
SOM (g kg^{-1})	15 dpi	119.25 ± 4.55aA	122.28 ± 1.52aA	125.31 ± 3.03aA	113.18 ± 4.61aA	112.42 ± 3.48aA	113.18 ± 4.61aA
SOM (g kg^{-1})	30 dpi	102.41 ± 2.63aA	113.03 ± 1.52aA	106.96 ± 4.55aA	102.41 ± 6.02aA	109.23 ± 1.31aA	100.89 ± 1.52aA
SOM (g kg^{-1})	45 dpi	93.30 ± 5.26aA	97.85 ± 2.63aA	102.41 ± 4.55aA	87.99 ± 2.74aA	100.13 ± 5.26aA	97.85 ± 3.94aA

Note: Values are means ± SE (n = 3) and are expressed in the units shown in the Property column. Lowercase letters indicate significant differences among N0, N1, and N2 within the same inoculation treatment at the same sampling stage, based on one-way ANOVA followed by Tukey's HSD test ($P < 0.05$). Uppercase letters indicate significant differences between CK and BX under the same simulated N deposition level at the same sampling stage, based on Student's independent-samples t-tests ($P < 0.05$). CK, non-inoculated; BX, inoculated with *B. xylophilus*; N0, no simulated N deposition; N1, low-level simulated N deposition; N2, medium-level simulated N deposition; dpi, days post inoculation; SOM, soil organic matter; AP, available phosphorus.

Table S2. Relative abundances (%) of selected fungal taxa discussed in the manuscript

**Taxon**	**Level**	**CKN0**	**CKN1**	**CKN2**	**BXN0**	**BXN1**	**BXN2**
Ascomycota	Phylum	52.19 ± 0.05A	60.29 ± 4.16A	68.96 ± 0.35A	50.19 ± 6.15A	68.84 ± 2.82A	73.05 ± 7.34A
Mortierellomycota	Phylum	7.80 ± 0.12A	23.79 ± 1.71A	1.86 ± 0.25A	1.20 ± 0.16B	5.93 ± 0.09B	1.41 ± 0.07A
Basidiomycota	Phylum	11.08 ± 0.94A	7.20 ± 1.44A	2.95 ± 0.68B	5.29 ± 2.76A	4.12 ± 1.22A	8.38 ± 0.15A
Talaromyces	Genus	1.20 ± 0.02B	2.04 ± 0.86B	0.36 ± 0.08A	10.55 ± 0.45A	8.65 ± 1.17A	0.61 ± 0.53A
Apiotrichum	Genus	2.35 ± 0.12A	0.65 ± 0.48A	0.47 ± 0.10A	0.13 ± 0.01B	0.42 ± 0.01A	0.31 ± 0.06A
Aspergillus	Genus	3.33 ± 0.40A	3.81 ± 0.00A	1.30 ± 0.20A	0.44 ± 0.01B	0.74 ± 0.00B	0.17 ± 0.02B

Note: Values are means ± SE and are expressed as relative abundance (%). Uppercase letters indicate significant differences between CK and BX under the same simulated N deposition level, based on Student's independent-samples t-tests. Different uppercase letters within the same simulated N deposition level indicate significant differences at $P < 0.05$; the same uppercase letters indicate no significant difference. CK, non-inoculated; BX, inoculated with *B. xylophilus*; N0, no simulated N deposition; N1, low-level simulated N deposition; N2, medium-level simulated N deposition.

Table S3. Relative abundances (%) of selected bacterial taxa discussed in the manuscript

**Taxon**	**Level**	**CKN0**	**CKN1**	**CKN2**	**BXN0**	**BXN1**	**BXN2**
Actinobacteriota	Phylum	22.49 ± 0.27A	56.64 ± 0.32A	50.22 ± 3.84A	33.02 ± 4.07A	33.60 ± 5.73A	66.61 ± 0.95A
Proteobacteria	Phylum	18.29 ± 0.60A	13.49 ± 0.39A	20.04 ± 0.80A	13.88 ± 2.52A	16.51 ± 0.92A	13.48 ± 1.05B
Acidobacteriota	Phylum	22.72 ± 0.22A	10.06 ± 0.06A	4.91 ± 0.61A	11.18 ± 0.95B	4.13 ± 0.05B	4.19 ± 1.02A
Nocardioides	Genus	0.67 ± 0.01B	8.02 ± 0.40A	4.29 ± 0.12B	2.34 ± 0.36A	2.68 ± 0.03B	4.99 ± 0.01A
RB41	Genus	5.43 ± 0.08A	3.74 ± 0.09A	1.33 ± 0.34A	2.45 ± 0.33B	0.80 ± 0.11B	1.79 ± 0.65A
Nitrosospira	Genus	0.01 ± 0.00A	0.35 ± 0.00A	0.54 ± 0.18A	0.01 ± 0.01A	0.96 ± 0.58A	0.30 ± 0.15A

Note: Values are means ± SE and are expressed as relative abundance (%). Uppercase letters indicate significant differences between CK and BX under the same simulated N deposition level, based on Student's independent-samples t-tests. Different uppercase letters within the same simulated N deposition level indicate significant differences at $P < 0.05$; the same uppercase letters indicate no significant difference. CK, non-inoculated; BX, inoculated with *B. xylophilus*; N0, no simulated N deposition; N1, low-level simulated N deposition; N2, medium-level simulated N deposition.

Table S4. Relative abundances (%) of selected predicted bacterial KEGG level-3 functional categories.

Community	Functional group/category	Level	CKN0	CKN1	CKN2	BXN0	BXN1	BXN2
Bacteria	Transporters	KEGG level-3 functional category	5.66 ± 0.02A	5.95 ± 0.04A	5.92 ± 0.02A	5.73 ± 0.05A	6.02 ± 0.14A	5.91 ± 0.04A
Bacteria	Two-component system	KEGG level-3 functional category	3.17 ± 0.03A	2.64 ± 0.08A	2.59 ± 0.06A	2.77 ± 0.15A	2.64 ± 0.10A	2.48 ± 0.07A
Bacteria	DNA repair and recombination proteins	KEGG level-3 functional category	2.76 ± 0.01A	2.51 ± 0.04A	2.51 ± 0.04A	2.62 ± 0.08A	2.54 ± 0.05A	2.45 ± 0.04A
Bacteria	Ribosome biogenesis	KEGG level-3 functional category	1.73 ± 0.02A	2.39 ± 0.09A	2.31 ± 0.08A	2.06 ± 0.14A	2.32 ± 0.14A	2.45 ± 0.11A
Bacteria	Messenger RNA biogenesis	KEGG level-3 functional category	1.65 ± 0.03A	2.41 ± 0.11A	2.32 ± 0.09A	2.04 ± 0.16A	2.31 ± 0.15A	2.49 ± 0.12A
Bacteria	RNA degradation	KEGG level-3 functional category	1.63 ± 0.03A	2.39 ± 0.10A	2.30 ± 0.10A	2.01 ± 0.16A	2.29 ± 0.16A	2.47 ± 0.12A
Bacteria	Quorum sensing	KEGG level-3 functional category	1.46 ± 0.01A	1.83 ± 0.05A	1.79 ± 0.04A	1.64 ± 0.08A	1.81 ± 0.09A	1.86 ± 0.06A
Bacteria	Purine metabolism	KEGG level-3 functional category	1.79 ± 0.00A	1.66 ± 0.02A	1.69 ± 0.02A	1.76 ± 0.02A	1.71 ± 0.04A	1.64 ± 0.02A
Bacteria	ABC transporters	KEGG level-3 functional category	1.43 ± 0.02A	1.72 ± 0.04A	1.74 ± 0.03A	1.59 ± 0.07A	1.74 ± 0.08A	1.79 ± 0.04A
Bacteria	Nitrogen metabolism	KEGG level-3 functional category	0.55 ± 0.00A	0.57 ± 0.00A	0.58 ± 0.00A	0.57 ± 0.01A	0.57 ±0.01A	0.59 ± 0.00A

Note: Values are means ± SE and are expressed as relative abundance (%). Uppercase letters indicate significant differences between CK and BX under the same simulated N deposition level, based on Student's independent-samples t-tests. Different uppercase letters within the same simulated N deposition level indicate significant differences at $P < 0.05$; the same uppercase letters indicate no significant difference. CK, non-inoculated; BX, inoculated with *B. xylophilus*; N0, no simulated N deposition; N1, low-level simulated N deposition; N2, medium-level simulated N deposition.
